# Bootstrap Model-Based Constrained Optimization Tests of Indirect Effects

**DOI:** 10.3389/fpsyg.2019.02989

**Published:** 2020-01-20

**Authors:** Davood Tofighi

**Affiliations:** University of New Mexico, Albuquerque, NM, United States

**Keywords:** indirect effect, mediation analysis, confidence interval, likelihood ratio test, constrained optimization

## Abstract

In mediation analysis, conditions necessary for commonly recommended tests, including the confidence interval (CI)-based tests, to produce an accurate Type I error, do not generally hold for finite sample sizes and non-normally distributed model residuals. This is typically the case because of the complexity of testing a null hypothesis about indirect effects. To remedy these issues, we propose two extensions of the recently developed asymptotic Model-based Constrained Optimization (MBCO) likelihood ratio test (LRT), a promising new model comparison method for testing a general function of indirect effects. The proposed tests, semi-parametric and parametric bootstrap MBCO LRT are shown to yield a more accurate Type I error rate in smaller sample sizes and under various degrees of non-normality of the model residuals compared to the asymptotic MBCO LRT and the CI-based methods. We provide R script in the Supplemental Materials to perform all three MBCO LRTs.

In statistics, the goal of mediation analysis is to understand what underpins an observed phenomenon, such as racism or gender identity, and to explain what mechanisms (mediators) contribute to creating that phenomenon. Various models allow researchers to study how a randomized intervention can influence an outcome through one or more mediators (e.g., Cury et al., 2002; Deković et al., 2012; Bernier et al., 2017; Donnelly et al., 2018). For example, a researcher might use a general sequential, two-mediator model to test hypotheses about racial profiling. As part of such a study the researcher may hypothesize that at Time 1, perceived social dominance orientation (SDO) increases, at Time 2 perceived sexism increases, and at Time 3 perceived threats to gender identity increase when the covariates are controlled (Sanchez et al., 2017). Thus, under a set of correct specifications and no-confounder assumptions, the researcher can define the indirect effect as a product of coefficients along the mediation chain, β_1_β_2_β_3_ (VanderWeele, 2015). To illustrate this design, consider the general sequential two-mediator model shown in Figure 1.

**Figure 1:**
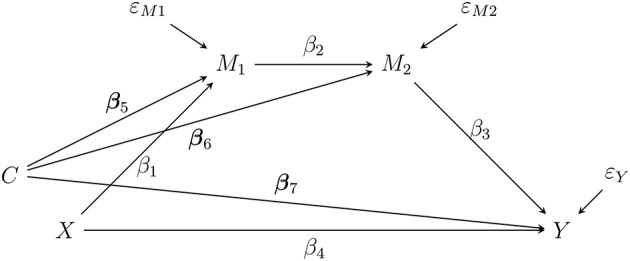
A sequential two-mediator chain as a path diagram. The effect of *X* (Racist Profile vs Control) and *Y* (threat to gender identity) is transmitted through a sequence of mediators measured over time. Note that *X* should be measured before *M*_1_ (social dominance orientation), *M*_1_ should be measured before *M*_2_ (perceived sexism), and *M*_2_ should be measured before *Y*. *C* (liking) is a covariate. ε_*M*1_, ε_*M*2_, and ε_*Y*_ are the residual terms.

However, when researchers want to test hypotheses about indirect effects, they have problems reporting their results statistically because the recommended methods have shortcomings that limit their usefulness (MacKinnon et al., 2002). Specifically, using confidence interval (CI) methods to test a null hypothesis about an indirect effect can produce too few or too many false-positives (i.e., erroneously concluding that an effect exists) and, therefore, inaccurate Type I error rates in practical cases, for example, when one of the coefficients is zero and *N* = 100 (MacKinnon et al., 2002; Williams and MacKinnon, 2008; Biesanz et al., 2010; Fritz et al., 2012; Falk and Biesanz, 2015; Koopman et al., 2015). All the CI-based methods yield an empirical Type I error rate that fluctuates across sample sizes, parameter values, and mediation models with varying complexities (e.g., a single-mediator model verses a sequential two-mediator model). In addition, problems with the CI-based tests of indirect effects tend to worsen in smaller sample sizes (*N* ≤ 100) or when the assumption of the multivariate normality of the residuals is violated (Taylor et al., 2008; Tofighi and Kelley, 2019). These limitations in testing methodology can lead researchers to use methods that would yield incorrect answers to important research questions, such as incorrectly concluding that an indirect effect exists for the model in Figure 1 when in fact it does not.

To address the shortcomings of the CI-based methods of testing an indirect effect, Tofighi and Kelley (in press) proposed a likelihood ratio test (LRT) test of general function of indirect effects, termed the asymptotic Model-based Constrained Optimization (MBCO) LRT, which is effective in a wide variety of mediation models. A simulation study showed that the asymptotic MBCO LRT yielded a more accurate Type I error rate than the commonly used methods of testing an indirect effect in that its empirical Type I error remained closer to the nominal significance level α in smaller sample sizes while the asymptotic MBCO LRT remained as powerful as the other tests. However, the simulation studies were conducted under the ideal condition of multivariate normality of the model residuals. As Tofighi and Kelley stated, because the asymptotic MBCO LRT was developed using the multivariate normal likelihood function, it is unknown whether the asymptotic MBCO LRT would maintain its accurate Type I error under moderate to severe degrees of multivariate non-normality of the model residuals, especially in smaller sample sizes. To address the issues with the asymptotic MBCO LRT, we offer two alternative bootstrap resampling methods based on the variants of the MBCO LRT that are theoretically more robust to the violation of the multivariate normality of the model residuals, and are expected to show superior statistical properties in smaller sample sizes: 1- parametric bootstrap MBCO and 2- semi-parametric bootstrap MBCO. We also conduct a simulation study comparing the Type I error rate and power of the two proposed bootstrap MBCO LRTs with the following recommended confidence interval (CI)-based methods in the literature (Tofighi and Kelley, 2019):

- percentile bootstrap (Bollen and Stine, 1990; Efron and Tibshirani, 1993; Shrout and Bolger, 2002; MacKinnon et al., 2004),
- profile likelihood (Folmer, 1981; Neale and Miller, 1997; Pawitan, 2001; Pek and Wu, 2015), and
- Monte Carlo (MacKinnon et al., 2004; Tofighi and MacKinnon, 2016).

In the Supplemental Material^1^, we provide an R (R Development Core Team, 2019) function that facilitates conducting the asymptotic, parametric bootstrap, and semi-parametric bootstrap MBCO LRTs.

## Issues With Currently Used Tests of Indirect Effect

Using a CI-based method might not produce the more accurate Type I error rate test because of two interwoven challenges (Tofighi and Kelley, 2019). First, the null hypothesis of zero indirect effect is a *composite* null hypothesis. To illustrate, consider testing the null hypothesis *H*_0_ : β_1_β_2_β_3_ = 0 in the sequential two-mediator model in Figure 1. This null hypothesis is composite because there are an infinite number of the null sampling distributions, the sampling distributions of the product of coefficients in which the null hypothesis of a zero indirect effect is true, rather than only one sampling distribution for which the null hypothesis is true. For instance, for any β_1_ = 0, β_2_ and β_3_ can take on any value but the null hypothesis of no indirect effect is still true and thus it is a composite null hypothesis. Similarly, for any β_2_ = 0, β_1_ and β_3_ may take on any value but the null hypothesis of no indirect effect is still true. The main difficulty with the composite null hypothesis is that we do not know which null mediational process, if any, to use to estimate the null sampling distribution. Because we do not know which parameter is zero and what the values of non-zero βs are, we cannot estimate the null sampling distribution. Not having determined the null sampling distribution, we cannot build a correct critical region and calculate a *p*-value.

Second, to test this composite null hypothesis, one must find a *pivot* or pivotal test quantity such that the optimal properties of a test (e.g., accuracy of the Type I error rate) holds true. A pivot is a random variable whose sampling distribution remains the same across different parameter values (DeGroot and Schervish, 2012). Currently used methods of testing an indirect effect, including CI-based tests, use the maximum likelihood estimator (MLE) of the indirect effect, such as ${\hat{\beta }}_{1}{\hat{\beta }}_{2}{\hat{\beta }}_{3}$, that is not a pivot. As a result, the null sampling distribution does vary as a function of the unknown parameters and across different mediation models. As previously indicated, this is important because the null sampling distribution must be first estimated to build a proper critical region and compute an accurate *p*-value.

## Bootstrap MBCO

In this section, we provide some background about the sequential two-mediator model in Figure 1 (Sanchez et al., 2017). The following equations are used to estimate the sequential two-mediator model:

(1) $$\begin{array}{l} M_{1}=\beta_{0,M1}+\beta_{1}X+\beta_{7}C+\varepsilon_{M1}\\ M_{2}=\beta_{0,M2}+\beta_{2}M_{1}+\beta_{4}X+\beta_{8}C+\varepsilon_{M2}\\ \;Y=\beta_{0,Y}+\beta_{3}M_{2}+\beta_{5}M_{1}+\beta_{6}X+\beta_{9}C+\varepsilon_{Y} \end{array}$$

where β_1_ is the effect of the independent variable (*X* = 0, control, 1, racism) on social dominance orientation (SDO; *M*_1_) controlling for covariate Liking (*C*); β_2_ is the effect of SDO on Perceived Sexism (*M*_2_) controlling for *X* and *C*; β_3_ is the effect of Perceived Sexism on Gender Stigma (*Y*) controlling for *X*, *M*_1_, and *C*. β_0,*M*1_, β_0,*M*2_, β_0,*Y*_ are the intercepts; β_7_ to β_9_ quantify the effect of *C* on the endogenous variables; and εs are the residuals. More succinctly, we can use matrix notation to describe assumptions about the model. Let $\epsilon ={\left(\varepsilon_{X},\varepsilon_{M1},\varepsilon_{M2},\varepsilon_{Y}\right)}^{\text{T}}$, where the superscript “T” denotes transpose operation. We assume that the vector of the residuals have a multivariate normal distribution with the 4 × 1 mean vector of zero and the covariance matrix **Σ**_ϵ_:

$$\Sigma_{\epsilon }=\text{diag}\left(\sigma_{X}^{2},\sigma_{M_{1}}^{2},\sigma_{M_{2}}^{2},\sigma_{Y}^{2}\right),$$

where diag denotes a diagonal matrix where the main diagonal elements are the residual variance and off-diagonal elements are zero. Later, we will use a model implied 4 × 4 covariance matrix, denoted by **Σ**.

### Asymptotic Model-Based Constrained Optimization LRT

In this section, we discuss a newly developed test of any smooth function of indirect effect termed asymptotic MBCO LRT (Tofighi and Kelley, in press). First, we formulate a general hypothesis testing framework about the indirect effect. Let $M_{Full}\left(\Theta \right)$ denote a full (hypothesized) mediation model and **Θ** denote the model parameter space. In the structural equation model (SEM) framework, **Θ** = {***β***, **Ψ**}, where ***β*** is a set of all the free regression (path) coefficients and **Ψ** is a set containing the unique elements of the covariance matrices of residual terms. While the elements in ***β*** may take on any real values, the variance terms in **ψ** have a lower bound of zero. For example, if $M_{Full}\left(\Theta \right)$ denotes the sequential two-mediator mediation model in Figure 1, then **Θ** contains all the regression coefficients (i.e., βs) and three residual variances for the endogenous variables. We next define a hypothesis testing framework to test a general function of indirect effect as follows:

(2) $$\begin{array}{l} H_{0}:\theta \in \Theta_{0}\\ \begin{array}{c} H_{1}:\theta \in \Theta_{1}, \end{array} \end{array}$$

where **θ** is a vector of the mediation model parameters and **Θ**_0_ denotes the null parameter space, which is a subset of the full model parameter space **Θ**. For example, for the two-mediator mediation model, the null parameter space contains all elements of **Θ** for which the indirect effect β_1_β_2_β_3_ is zero. In addition, the alternative parameter space, denoted by **Θ**_1_, contains all the parameter values that are in **Θ** but not in **Θ**_0_: **Θ**_1_ = **Θ** \ **Θ**_0_, where the symbol “\” denotes the set difference operation^2^.

As mentioned before, because the MLE of an indirect effect is not a pivot to test zero indirect effect, determining a unique null distribution is not feasible. There exist an infinite number of null sampling distributions whose parameters would be within the null parameter space in (2). To remedy this problem, we recast the null hypothesis testing in (2) in terms of the model comparison framework. That is, we use a model comparison framework to test a general function of indirect effect. In the model comparison framework, we define two models under as follows:

(3) $$\begin{array}{l} H_{0}:M_{0}\left(\Theta_{0}\right)\\ \begin{array}{c} H_{1}:M_{Full}\left(\Theta \right), \end{array} \end{array}$$

where $M_{0}\left(\Theta_{0}\right)$, termed *null* model, is estimated over the null parameter space. Note that in the model comparison framework, we use $M_{Full}\left(\Theta \right)$, termed *full* model, that is the mediation model estimated without being subject to the restrictions imposed in either the null or the alternative parameter space in (2). It turns out that in the model comparison framework, one can use the full parameter space **Θ** instead of the more restricted alternative parameter space^3^ (Wasserman, 2004). The model comparison framework in (3) is more convenient for the composite hypothesis testing because defining the alternative hypothesis parameter space is more involved. Note that $M_{Full}\left(\Theta \right)$ is basically estimating the model without any additional restriction beyond theory-driven ones already posited by the researcher. For example, $M_{Full}$ is the two-mediator sequential mediation model in Figure 1.

To estimate the null model, it is important to carefully define the null parameter space. We define the null parameter space as follows:

(4) $$\begin{array}{l} \Theta_{0}=\left\{\forall \beta \in \Theta :g\left(\beta \right)\geq 0\right\}, \end{array}$$

where *g*(***β***) is a smooth, scalar function of the path coefficients in the model and possibly a non-zero constant. Also, it should be noted the symbol “≥” indicates that we can test a one-sided as well as a two-sided null hypothesis, although we only discuss a two-sided null hypothesis. For example, to test whether the indirect effect β_1_β_2_β_3_ is zero, the null parameter space is defined as **Θ**_0_ = {∀***β*** ∈ **Θ**:*g*(***β***) = β_1_β_2_β_3_ = 0}, where β_1_β_2_β_3_ = 0 is a non-linear equality constraint.

Now, a critical question is how to estimate the null model. Herein lies the innovation of the asymptotic MBCO LRT. The null model is formulated as a non-linear constrained optimization problem in which the goal is to maximize the likelihood function *L*(·) subject to the non-linear constraint *g*(β) = 0:

(5) $$\begin{array}{l} \text{Maximize }L\left(\theta |y\right)\\ \begin{array}{c} \text{subject to }g\left(\beta \right)\geq 0 \end{array} \end{array}$$

where *g*(***β***) is a smooth, scalar function of the path coefficients in the model as in (4) and $y={\left(X,M_{1},M_{2},Y\right)}^{\text{T}}$ is a vector of the observed variables. Comparing the non-linear constraint in (5) with the null parameter space defined in (4), we can observe that the non-linear constraint in the optimization problem essentially ensures that the model parameter estimates lie within the null parameter in which the general function of model parameters satisfies the constraint.

Now, a brief overview of the conducting the asymptotic MBCO LRT procedure follows.

(6) $$\begin{array}{l} {\text{T}}_{MBCO}=-2\text{ log}\frac{L\left({\hat{\beta }}_{0},{\hat{\psi }}_{0}|\text{y}\right)}{L\left(\hat{\beta },\hat{\psi }|\text{y}\right)} \end{array}$$

where ${\hat{\beta }}_{0}$ and ${\hat{\psi }}_{0}$ are the MLEs of the null model, and $\hat{\beta }$ and $\hat{\psi }$ are the MLEs of the full model. If the likelihood of the null model over **Θ**_0_ is sufficiently small compared to that of the full model over **Θ**, the null hypothesis is rejected. Thus, the *T*_*MBCO*_ has the rejection region *C*_α_ = {**y** : *T*_*MBCO*_ ≤ *c*_0_}, where *c*_0_ is a critical value chosen so that the *size* of the *T*_*MBCO*_ is less than or equal to α— size of a test is the probability that the test falsely rejects the null hypothesis. To compute the critical value *c*_0_, we need to determine the sampling distribution of *T*_*MBCO*_.

We formulated *T*_*MBCO*_ such that it is minus twice the log function of the likelihood ratio. Because *T*_*MBCO*_ is a likelihood ratio test, it has an asymptotic limiting χ^2^ distribution (Cox and Hinkley, 2000, pp. 322–323; Wilks, 1938). More formally, when certain regularity conditions are met (See Silvapulle and Sen 2001, p. 146), the null hypothesis in (2) holds, and the sample size is large,

(7) $$\begin{array}{l} {\text{T}}_{MBCO}\sim \chi^{2}\left(\nu \right), \end{array}$$

where ν denotes the degrees of freedom that equals the difference in the number of the free parameters between the null and full models, respectively. A critical result is that the limiting chi-square distribution does not depend on the unknown model parameters. In other words, asymptotically, the proposed MBCO LRT is a pivot. One of the regularity conditions is that the null parameter values in (2) may not be on the boundary of the parameter space. Because the non-linear constraint in (4) is defined by a smooth function of regression coefficients that may take on any real values, the parameter values defined in (4) do not lie on the boundary of the null parameter space.

### Bootstrap MBCO LRT

In computing the asymptotic MBCO LRT, we made several distributional and large sample assumptions. First, the likelihood function in (5) assumes that the model residuals have a multivariate normal distribution. Second, the MBCO LRT has a large sample chi-square distribution. When these assumptions are not met, that is when the model residuals do have a multivariate normal distribution, or in smaller sample sizes, it is unclear if the asymptotic MBCO LRT has a chi-square distribution. In these situations, bootstrapping techniques can be appealing as they tend to rely less on the assumption of the multivariate normality and can perform better in smaller sample sizes (Efron and Tibshirani, 1993).

For bootstrap hypothesis testing, a major requirement is that bootstrap samples should be drawn from the sample that is transformed to match the null model (Beran, 1988). Resampling from the transformed sample would then mimic drawing from the null sampling distribution. If the bootstrap samples are not drawn from the sample with an adjustment to conform to the null model, the sampling distribution is likely to differ from the null distribution. Such resampling is sometimes called “naive” bootstrap (Bollen and Stine, 1992). Thus, a challenge for all the bootstrapping techniques discussed below is how to conduct resampling from the transformed sample so that the bootstrap resampling distribution matches the null distribution. Next, we discuss two bootstrap extensions of the MBCO LRT that would correctly perform resampling under the null hypothesis.

### Parametric Bootstrap MBCO LRT

In the parametric bootstrap, a mediation model is first fitted to data using an estimation method such as the maximum likelihood (ML). The implied mean vector and covariance matrix of the fitted model are used to replace the true mean and covariance matrix of distribution of the data. If we assume that data has a multivariate normal distribution, then repeated samples are drawn from the distribution with the mean vector and covariance matrix estimated from the fitted model. Each data set is used to estimate a mediation model and the quantities of interest (e.g., indirect effect) resulting in a parametric bootstrap sampling distribution for the indirect effect. It should be noted that the parametric bootstrap procedure we just described does not take into account the null model, and thus it is a “naive” parametric bootstrap. As previously indicated, before using bootstrapping for hypothesis testing, we must ensure that the sampling distribution is drawn from the model under the null hypothesis. That is, for the parametric bootstrap, the bootstrap samples must be drawn from the fitted null model that satisfies constraints imposed under the null hypothesis.

Because the asymptotic MBCO LRT uses a model comparison framework, we can extend the test to accommodate parametric bootstrap hypothesis testing that would satisfy the null hypothesis. Note that in the asymptotic MBCO LRT, the null model is estimated under the null hypothesis. We use the fitted null model estimates to draw samples from the estimated null distribution. That is, we sample from a multivariate normal distribution whose parameters are estimated from the fitted null model. We propose the following steps to compute parametric bootstrap MBCO LRT:

1. Draw a random sample from the multivariate normal distribution whose mean and covariance matrix are estimated from the fitted null mediation model.
2. Estimate the null and full mediation for the sample and compute the MBCO LRT statistic.
3. Repeat the previous steps *R* times, which would result in *R* bootstrap samples from the null distribution of LRTs.
4. Compute *p*-value as the proportion of the bootstrapped LRTs greater than original LRT value, which is computed from the asymptotic MBCO LRT.

### Semi-parametric Bootstrap MBCO LRT

In semi-parametric bootstrap for hypothesis testing, we draw samples from the transformed sample data to conform to the model under the null hypothesis (Beran and Srivastava, 1985; Beran, 1988; Bollen and Stine, 1992). As mentioned before, the idea of resampling from a transformed sample that supports the null hypothesis is essential in estimating the null sampling distribution of MBCO LRT. For the semi-parametric bootstrap MBCO LRT, this is done by first transforming the original sample and then drawing repeated random draws with replacement from the transformed data.

Before proceeding, we discuss a few matrix algebra results essential in understanding the algorithm for the semi-parametric bootstrap MBCO LRT. Below, we show how we use matrix transformation to make the original sample data more compatible with the data as if the data were generated under the null hypothesis. First, we explain Cholesky transformation. A positive-definite matrix **M**, such as a covariance matrix, can be uniquely factored into a product **M** = **U**^T^**U**, where **U**, called a Cholesky matrix, is an upper triangular matrix with positive diagonal entries. To illustrate, consider the sample covariance matrix.

$$\begin{array}{l} \text{S}=\frac{1}{N-1}\overset{N}{\underset{i=1}{\sum }}\left(y_{i}-\bar{y}\right){\left(y_{i}-\bar{y}\right)}^{\text{T}}, \end{array}$$

where $y_{i}={\left(X_{i},M_{1i},M_{2i},Y_{i}\right)}^{\text{T}}$ and $\bar{y}$ be the 4 × 1 vector of the sample means for *N* observations. Using Cholesky factorization on the sample covariance matrix, we have $\text{S}={\text{U}}_{\text{S}}^{\text{T}}{\text{U}}_{\text{S}}$. Using the sample covariance factorization, we factorize the inverse of the sample covariance as follows:

(8) $$\begin{array}{l} {\text{S}}^{-1}={\left({\text{U}}_{\text{S}}^{\text{T}}{\text{U}}_{\text{S}}\right)}^{-1}={\text{U}}_{\text{S}}^{-1}{\text{U}}_{\text{S}}^{-\text{T}} \end{array}$$

We also use Cholesky factorization on the fitted null model implied covariance matrix as follows:

(9) $${\hat{\Sigma }}_{0}={\text{U}}_{{\hat{\Sigma }}_{0}}^{\text{T}}{\text{U}}_{{\hat{\Sigma }}_{0}}$$

The algorithm for the semi-parametric bootstrap MBCO LRT is as follows:

1. Transform data as follows: $y^{*}={\text{U}}_{{\hat{\Sigma }}_{0}}^{\text{T}}{\text{U}}_{\text{S}}^{-\text{T}}$, where y^*^ is the transformed data and ${\text{U}}_{\text{S}}^{-\text{T}}$ and ${\text{U}}_{{\hat{\Sigma }}_{0}}^{\text{T}}$ are computed in (8) and (9), respectively.
2. Take sample of size *N* with replacement from the transformed data y^*^, fit the null and full model to resampled data, and compute MBCO LRT.
3. Repeat Step 2 *R* times, which would result in the null sampling distribution of MBCO LRT statistic.
4. Compute *p*-value as the proportion of the bootstrapped LRTs greater than original LRT value, which is computed from the asymptotic MBCO LRT.

Below, we show that the covariance matrix of the transformed data equals the implied covariance matrix of the fitted null model. That is, we show that the covariance matrix of the transformed data equals the covariance matrix of the fitted null model: var(y^*^) = ${\hat{\Sigma }}_{0}$.

$$\begin{array}{l} \text{var}(y^{*})=\text{ var }\left[\left({\text{U}}_{{\hat{\Sigma }}_{0}}^{\text{T}}{\text{U}}_{\text{S}}^{-\text{T}}\right)y\right]=\left({\text{U}}_{{\hat{\Sigma }}_{0}}^{\text{T}}{\text{U}}_{\text{S}}^{-\text{T}}\right)\text{ var(}y)\;\left({\text{U}}_{\text{S}}^{-1}{\text{U}}_{{\hat{\Sigma }}_{0}}\right)\\ =\left({\text{U}}_{{\hat{\Sigma }}_{0}}^{\text{T}}{\text{U}}_{\text{S}}^{-\text{T}}\right){\text{U}}_{\text{S}}^{\text{T}}{\text{U}}_{\text{S}}\left({\text{U}}_{\text{S}}^{-1}{\text{U}}_{{\hat{\Sigma }}_{0}}\right)={\text{U}}_{{\hat{\Sigma }}_{0}}^{\text{T}}\left({\text{U}}_{\text{S}}^{-\text{T}}{\text{U}}_{\text{S}}^{\text{T}}\right)\left({\text{U}}_{\text{S}}{\text{U}}_{\text{S}}^{-1}\right){\text{U}}_{{\hat{\Sigma }}_{0}}\\ \;={\text{U}}_{{\hat{\Sigma }}_{0}}^{\text{T}}{\text{U}}_{{\hat{\Sigma }}_{0}}={\hat{\Sigma }}_{0} \end{array}$$

## Review of Existing Methods

In this section, we briefly discuss currently recommended CI-based methods of testing indirect effects that have been recommended for both normality and non-normality of the model residuals. We follow the recommendation that different sets of methods may be used to test and to build a CI for indirect effect as these two goals might not overlap (Tofighi and Kelley, 2019):

- percentile bootstrap (Bollen and Stine, 1990; Efron and Tibshirani, 1993; Shrout and Bolger, 2002; MacKinnon et al., 2004),
- profile likelihood (Folmer, 1981; Neale and Miller, 1997; Pawitan, 2001; Pek and Wu, 2015), and
- Monte Carlo (MacKinnon et al., 2004; Tofighi and MacKinnon, 2016).

We do not consider bias-corrected (BC) bootstrap as it has been shown to yield inflated Type I error rates (Biesanz et al., 2010; Koopman et al., 2015).

### Percentile Bootstrap CI

In non-parametric bootstrap, *R* random samples of size *N* are drawn with replacement from the original data set (Efron and Tibshirani, 1993). Each observation has the same chance of the being selected in each draw. A mediation model is fitted to each sample resulting in a bootstrap sample with *R* estimates of model parameters and quantities of interest such as indirect effects. The percentile method uses α/2 and 1 − α/2 quantiles of the bootstrap sample to compute 100(1 − α)% CI for the population indirect effect.

### Profile Likelihood CI

In its simplest form, the profile-likelihood approach produces a CI for a single parameter using the profile likelihood function (Folmer, 1981; Neale and Miller, 1997; Pawitan, 2001; Cheung, 2007; Pek and Wu, 2015). The profile-likelihood function for the sequential two-mediator model, *L*(***θ***|*ı*, **y**), is computed by assuming that indirect effect, denoted by *ı* = β_1_β_2_β_3_, is a known quantity. That is, the indirect effect is fixed at a specific value, and thus *ı* is said to be profiled out of the likelihood function. The profile-likelihood function is treated as any likelihood function except that the quantity of interest (i.e., indirect effect) is assumed to be known. Thus, the profile-likelihood function depends on the fixed, but unknown values of the quantity of interest.

Next, we compare the log of the maximized profile-likelihood function and the maximized full model likelihood function; we have

$$\begin{array}{l} -2\text{ log}\frac{L({\hat{\theta }}_{\text{prof}}|ı,\text{y})}{L(\hat{\theta }|\text{y})} \end{array}$$

where $L\left({\hat{\theta }}_{\text{prof}}|1,y\right)$ and $L\left(\hat{\theta }|y\right)$ are the maximum profile-likelihood and full likelihood functions, respectively; ${\hat{\theta }}_{\text{prof}}$ and $\hat{\theta }$ are the profile-likelihood and full MLE of the model parameters, respectively. Asymptotically, the difference in twice negative maximum log-likelihood of the two functions has a chi-square distribution with one degree of freedom (Cheung, 2007), which is the difference in the number of the parameters between the profile likelihood and full likelihood functions. Then, the lower and upper limits for the 100 (1-α)% profile-likelihood correspond to the minimum and maximum of all values of the indirect effect that satisfy the following equality:

$$\begin{array}{l} -2\left\{LL\left({\hat{\beta }}_{\text{prof}},{\hat{\psi }}_{\text{prof}}|ı,\text{y}\right)-LL\left(\hat{\beta },\hat{\psi }|\text{y}\right)\right\}=\chi_{\alpha }^{2}\left(1\right) \end{array}$$

where *LL* denotes the log of a likelihood function and $\chi_{\alpha }^{2}\left(1\right)$ denotes the upper α critical value of chi-square distribution with one degree of freedom.

### Monte Carlo CI

The Monte Carlo method is a general and flexible method that estimates the sampling distribution of a function of parameters such as indirect effects (Tofighi and MacKinnon, 2016). To compute a Monte Carlo 100(1 − α)% CI for the indirect effect β_1_β_2_β_3_, *R* random samples are drawn from a multivariate normal distribution whose mean vector equals the MLEs of the coefficients β_1_, β_2_, and β_3_, and whose covariance matrix equals the covariance matrix of the MLEs. The product of the Monte Carlo random draws for the parameters β_1_, β_2_, and β_3_ yields a Monte Carlo sample of *R* random draws from the sampling distribution of the indirect effect. The mean and standard deviation of the Monte Carlo sample of the indirect effect are the estimate of mean and standard error of the indirect effect, respectively. The Monte Carlo 100(1 − α)% CI equals [*q*_α/2_, *q*_1−α/2_], where *q*_α/2_, *q*_1−α/2_ are α/2 and 1 − α/2 quantiles of the Monte Carlo sample of the indirect effect.

## Simulation Study

We conducted a simulation study to assess the Type I error and statistical power of the two proposed bootstrap extensions of the MBCO LRT and currently recommended methods of testing indirect effect across different sample sizes, parameter values, and distributional assumptions of model residuals. We used the sequential two-mediator model in Figure 1 to generate data and test the hypothesis: *H*_0_ : β_1_β_2_β_3_ = 0. The simulation was designed to answer the following questions: a) Does the asymptotic MBCO LRT show accurate Type I error rate when the assumption of multivariate normality of the model residuals is violated?— this condition has not been studied before. (b) Are the two proposed bootstrap MBCO LRTs more accurate and powerful than the asymptotic MBCO LRT as well as the CI-based tests? (c) Do the proposed bootstrap MBCO LRTs perform better than the asymptotic MBCO LRT and CI-based tests in terms of Type I error and power for the conditions of the non-normal residuals and in smaller sample sizes?

Based on similar previous simulation studies (Williams and MacKinnon, 2008; Falk and Biesanz, 2015; Tofighi and Kelley, 2019), we manipulated the following four factors: (a) semi-partial *R*^2^s, (b) sample size (*N*), (c) methods of testing indirect effect, and (d) multivariate distributions. For the semi-partial *R*^2^, we used Cohen (1988)'s general guideline, *R*^2^ = 0, 0.02 (“small”), 0.13 (“medium”), and 0.26 (“large”), and computed the corresponding model parameters: β = 0, 0.14, 0.39, and 0.59 (Thoemmes et al., 2010). We chose the following sample sizes that were used in previous simulation studies: 50, 100, 200, and 500. Because in our preliminary simulation study, the sample size greater than 500 did not produce discerning differences in the performance of the methods, we did not investigate larger sample sizes. We considered the sample size of 50 as a lower bound for most psychological studies. The third factor was the six methods of testing an indirect effect:

- asymptotic MBCO LRT,
- parametric bootstrap MBCO LRT,
- semi-parametric bootstrap MBCO LRT,
- percentile non-parametric bootstrap,
- profile-likelihood CI, and
- Monte Carlo CI.

For the fourth factor, we considered three levels of multivariate distributions for the model residuals, one multivariate normal and two multivariate non-normal distributions (Tofighi and Kelley, 2019). The multivariate normal distribution condition was considered an ideal situation and was used as a benchmark to gauge the performance of the two newly developed bootstrap MBCO LRTs as well as the existing methods. We considered two multivariate non-normal distributions, “moderate” and “severe” non-normality conditions (Finch et al., 1997). The multivariate non-normal distributions were generated such that the marginal univariate skewness and kurtosis of the residuals were 2 and 7 for moderate and 3 and 21 for severe conditions, respectively (Vale and Maurelli, 1983).

We used a full factorial design for each simulation study. We generated 1,000 independent data sets for each combination of the design factors using the simulateData function of the lavaan package Version 6.3 (Rosseel, 2012) that accommodates the generation of multivariate normal and non-normal distribution. We conducted the asymptotic, parametric bootstrap, and semi-parametric bootstrap MBCO LRT, as well as the profile-likelihood method CI in OpenMx Version 2.13.2 with Sequential Least-Squares Quadratic Programming (SLSQP) optimizer (Neale et al., 2016; Zahery et al., 2017). We used lavaan for the percentile bootstrap CI. We used 1,000 samples for the bootstrap methods. For the Monte Carlo CI, we first estimated the mediation model in lavaan and then used the RMediation package Version 1.1.4 (Tofighi and MacKinnon, 2011, 2016) to compute CIs with 100,000 random draws.

### Results

The outcomes of the simulation study were the empirical (observed) Type I error and power. The Type I error rate was the proportion out of the 1,000 replications that a method incorrectly rejected the null hypothesis of zero indirect effect. The empirical power was the proportion of the 1,000 replications that a test correctly detected a non-zero indirect effect. We considered a test to have an accurate Type I error rate if its empirical Type I error rate fell within Bradley (1978)'s interval of 0.025 and 0.075. If the Type I error rate exceeded the upper limit, the test was liberal; otherwise, the test was conservative.

#### Type I Error

**Figure 2:**
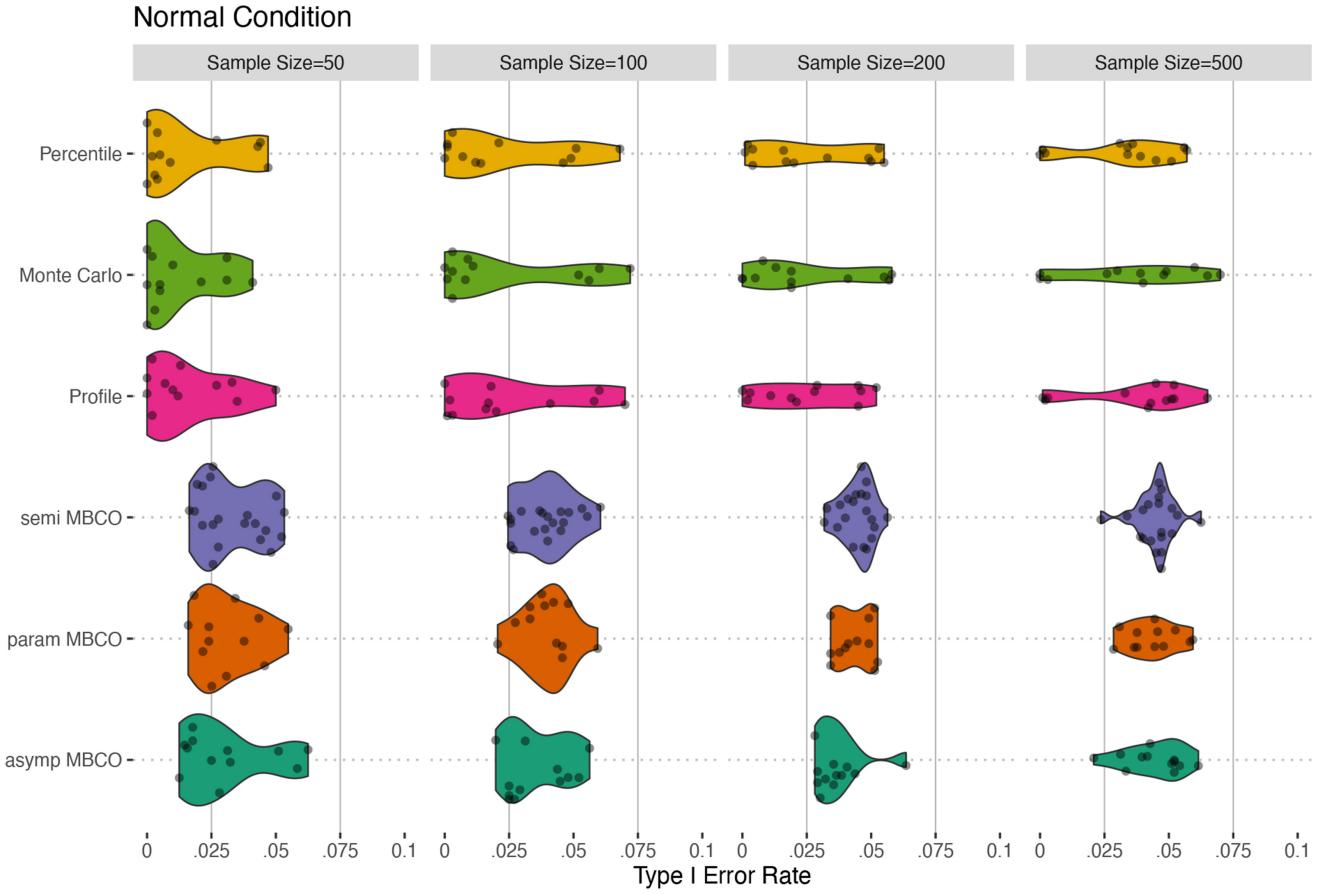
Type I error for normal conditions for testing an indirect effect, β_1_β_2_β_3_, in a two-mediator sequential chain. Horizontal solid lines show the limits of Bradley (1978)'s interval of 0.025 and 0.075 for α = 0.05. asymp MBCO, asymptotic MBCO LRT; param MBCO, parametric bootstrap MBCO LRT; semi MBCO, semi-parametric bootstrap MBCO LRT; Profile, Profile-Likelihood CI; Monte Carlo, Monte Carlo CI; Percentile, Percentile non-parametric bootstrap.

**Figure 3:**
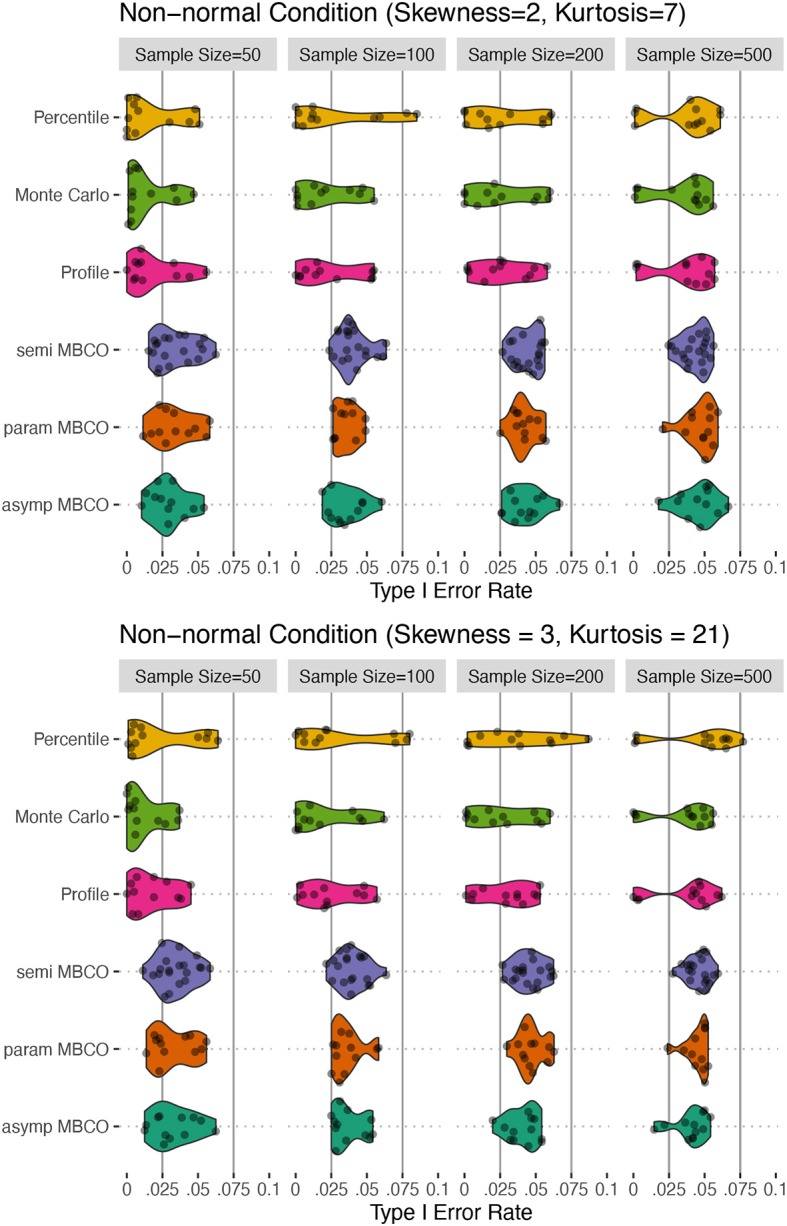
Type I error for non-normality conditions for testing an indirect effect, β_1_β_2_β_3_, in a two-mediator sequential chain. Horizontal solid lines show the limits of Bradley (1978)'s interval of 0.025 and 0.075 for α = 0.05. asymp MBCO, asymptotic MBCO LRT; param MBCO, parametric bootstrap MBCO LRT; semi MBCO, semi-parametric bootstrap MBCO LRT; Profile, Profile-Likelihood CI; Monte Carlo, Monte Carlo CI; Percentile, Percentile non-parametric bootstrap.

Figures 2, 3 show the violin plots for the Type I errors of the six methods across different sample sizes and the distribution of the model residuals. For the normal distribution condition, in general, the MBCO LRTs showed more accurate Type I error rates than did the CI-based tests. Specifically, when *N* = 50, the MBCO LRTs showed conservative Type I error rates that were more accurate (less conservative) than those of the CI-based tests. As the sample size increased, parametric and semi-parametric MBCO LRTs were the most accurate followed by the asymptotic MBCO LRTs. The CI-based tests also became more accurate as the sample size increased; however, they showed less accuracy than the MBCO LRTs.

For the non-normal conditions, a similar trend was observed. In general, the MBCO LRTs showed more accurate Type I error rates compared to the CI-based tests. For both non-normality conditions, in general, semi-parametric and parametric bootstrap MBCO LRTs were the most accurate followed by the asymptotic MBCO LRT. The CI-based methods were less accurate than the MBCO LRTs. The profile-likelihood and Monte Carlo methods showed similar accuracy that were more conservative than all the tests. For *N* ≥ 100, the percentile bootstrap CI showed instances of inflated Type I error rate beyond the upper limit of the Bradley (1978)'s interval.

#### Power

Tables 1, 2 show a subset of results where β_1_ = β_2_ = β_3_ for the power of the six methods across different sample sizes and the distribution of the model residuals. For the normal condition, difference in power between the six methods did not exceed 0.06 except in two cases between the percentile bootstrap and profile-likelihood method: medium effect size (β_1_ = β_2_ = β_3_ = 0.39) and *N* = 50 where the difference was 0.11 and small effect size and *N* = 500 where the difference was 0.08. In both cases, the profile-likelihood method showed higher power. For the non-normal condition where skewness = 2 and kurtosis = 7, the two largest power differences (0.12 and 0.1) occurred when (a) *N* = 50 and effect size was medium between the asymptotic MBCO LRT (0.47) and Monte Carlo CI (0.35), and (b) when effect size was small and *N* = 500, between semi-parametric MBCO LRT (0.73) and Monte Carlo CI (0.63). For the non-normal conditions where skewness = 3 and kurtosis = 21, the maximum difference in power of 0.12 occurred in two conditions between the percentile and Monte Carlo method: (a) when the effect size was small and *N* = 500, and (b) when effect size was medium and *N* = 50. Across all the distribution conditions, when *N* ≥ 200 and the effect size was medium or large, and when *N* = 100 and the effect size was large, all the methods had a power of 1.

**Table 1:** Empirical power for six methods of testing indirect effect for normal data condition.

**β_1_ = β_2_ = β_3_**	**N**	**asymp MBCO**	**param MBCO**	**semi MBCO**	**Profile**	**Monte Carlo**	**Percentile**
0.14	50	0.01	0.01	0.01	0.01	0	0
	100	0.03	0.03	0.03	0.02	0.01	0.01
	200	0.13	0.15	0.15	0.16	0.1	0.12
	500	0.68	0.7	0.72	0.73	0.68	0.65
0.36	50	0.46	0.43	0.43	0.49	0.41	0.38
	100	0.9	0.89	0.89	0.89	0.88	0.86
	200	1	1	1	1	1	1
	500	1	1	1	1	1	1
0.48	50	0.89	0.88	0.88	0.88	0.86	0.85
	100	1	1	1	1	1	1
	200	1	1	1	1	1	1
	500	1	1	1	1	1	1

*The β coefficients values 0.14, 0.36, and 0.48 correspond to the R^2^ = 0.02 (“small”), 0.13 (“medium”), and 0.26 (“large”) according to Cohen (1988)'s general guideline, respectively. asymp MBCO, asymptotic MBCO LRT; param MBCO, parametric bootstrap MBCO LRT; semi MBCO, semi-parametric bootstrap MBCO LRT; Profile, Profile-Likelihood CI; Monte Carlo, Monte Carlo CI; Percentile, Percentile non-parametric bootstrap*.

**Table 2:** Empirical power for six methods of testing indirect effect for non-normal data conditions.

**β_1_ = β_2_ = β_3_**	**N**	**asymp MBCO**	**param MBCO**	**semi MBCO**	**Profile**	**Monte Carlo**	**Percentile**
**Non-normal Condition (skewness** **=** **2, Kurtosis** **=** **7)**							
0.14	50	0.01	0.01	0.01	0.01	0.01	0
	100	0.03	0.03	0.03	0.03	0.01	0.01
	200	0.16	0.16	0.16	0.15	0.1	0.08
	500	0.7	0.7	0.73	0.66	0.63	0.65
0.36	50	0.47	0.44	0.44	0.43	0.35	0.36
	100	0.9	0.89	0.9	0.85	0.86	0.87
	200	1	1	1	1	1	1
	500	1	1	1	1	1	1
0.48	50	0.88	0.88	0.88	0.82	0.78	0.85
	100	1	1	1	0.99	1	1
	200	1	1	1	1	1	1
	500	1	1	1	1	1	1
**Non-normal Condition (Skewness** **=** **3, Kurtosis** **=** **21)**							
0.14	50	0.01	0.01	0	0.01	0	0
	100	0.02	0.03	0.03	0.03	0.02	0.02
	200	0.14	0.14	0.15	0.14	0.1	0.13
	500	0.7	0.69	0.7	0.64	0.61	0.73
0.36	50	0.45	0.43	0.43	0.38	0.35	0.47
	100	0.91	0.9	0.89	0.83	0.83	0.91
	200	1	1	1	1	1	1
	500	1	1	1	1	1	1
0.48	50	0.89	0.88	0.87	0.8	0.82	0.9
	100	1	1	1	0.98	0.99	1
	200	1	1	1	1	1	1
	500	1	1	1	1	1	1

*The β coefficients values 0.14, 0.36, and 0.48 correspond to the R^2^ = 0.02 (“small”), 0.13 (“medium”), and 0.26 (“large”) according to Cohen (1988)'s general guideline, respectively. asymp MBCO, asymptotic MBCO LRT; param MBCO, parametric bootstrap MBCO LRT; semi MBCO, semi-parametric bootstrap MBCO LRT; Profile, Profile-Likelihood CI; Monte Carlo, Monte Carlo CI; Percentile, Percentile non-parametric bootstrap*.

## Discussion

In this article, we proposed two extensions of the recently developed asymptotic MBCO LRT, which is a model-comparison approach to testing a general function of indirect effect in mediation analysis. Although the asymptotic MBCO LRT yields more accurate Type I error rate compared to the recommended CI-based methods, its performance for smaller sample size and multivariate non-normal distributions of the model residuals had not previously been examined. Because the asymptotic MBCO LRT relies on the normal likelihood function and has an asymptotically chi-squared distribution, its accuracy is likely to suffer in smaller sample sizes, for multivariate non-normal model residuals, or both. To remedy these deficiencies, we first proposed two extensions of the MBCO LRTs: parametric and semi-parametric bootstrap MBCO LRT, which we showed to produce more accurate Type I error for smaller sample sizes as well as non-normal model residuals. Our proposed methods are based on the bootstrap technique that tend to make fewer distributional assumptions than does the asymptotic MBCO LRT. Thus, theoretically the proposed tests were expected to have better statistical properties for smaller sample sizes and non-normal model residuals.

A simulation study was conducted to assess and compare the Type I error and power of the two proposed tests to the asymptotic MBCO LRT and three recommend CI-based tests, percentile bootstrap, profile-likelihood, and Monte Carlo method. The simulation study results showed that

- the MBCO LRTs all were more accurate in terms of the empirical Type I error rate (i.e., the Type I error rate was closely matched or was closer to the nominal significance level of α) than the CI-based tests,
- semi-parametric and parametric bootstrap MBCO LRT in general yielded more accurate Type I error rate than the asymptotic MBCO LRT for the non-normal model residuals and in smaller sample sizes,
- all the MBCO LRTs were as powerful as the best CI-based tests, and
- the percentile bootstrap CI showed instances of the inflated Type I error rate when the model residuals had multivariate non-normal distributions.

While our simulation studies did not show a discernible difference in the performances of the parametric and semi-parametric MBCO LRT, the two methods differ in how they estimate a null sampling distribution of LRTs. The parametric and semi-parametric MBCO LRT use bootstrapping to estimate the null sampling distribution of LRTs. In the parametric method, we fit the null model to the data, and then use the estimated model parameters (mean vector and covariances matrix) to simulate *R* parametric bootstrap samples from a multivariate normal distribution. We then fit the null and full model to each bootstrap sample, and compute MBCO LRT for each bootstrap sample that results in *R* samples from a null sampling distribution of LRT. In the semi-parametric MBCO LRT, however, we do not make a parametric assumption about the estimated parameters of the null model. We use the null model estimates to transform the original data and then resample from the transformed data to compute a null sampling distribution of LRT. Thus, the semi-parametric bootstrap MBCO LRT makes fewer assumptions.

While we recommend that researchers report a CI to illustrate a range of plausible values and uncertainty about an indirect effect, we do not advise that CIs be used to test an indirect effect (Tofighi and Kelley, 2019). Instead, we recommend that researchers employ one of the MBCO LRTs based on the distribution of the model residuals. If multivariate normality can be reasonably assumed, then one may use any of the MBCO LRTs. However, if the multivariate normality assumption is untenable, then we recommend researchers conduct both the semi-parametric and parametric bootstrap MBCO LRT and compare the results. If both methods agree, then one could report either test result. If the methods do not agree, then we recommend that researchers report the semi-parametric MBCO LRT because it makes fewer assumptions.

When testing an indirect effect, researchers should first examine the underlying assumptions necessary to enhance support for a causal claim about an indirect effect. Even in a randomized mediation model, in which participants are randomly assigned to treatment and control groups, making causal claims about an indirect effect requires a strong support for the no-omitted-confounder assumption (VanderWeele, 2015). That is, there should not be a variable omitted from the model that would confound (influence) either the mediator or the outcome variables, given the independent variable and covariates. If a researcher believes that not all confounders are included in the model, then a causal claim about the magnitude and existence of indirect effect needs to be qualified. Because the no-omitted confounder assumption is unstable, we recommend researchers conduct a sensitivity analysis to investigate the potential impact of omitted confounders on the inference about indirect effects (VanderWeele, 2015; Tofighi et al., 2019).

Several areas remain a topic of future study. One topic is the extension of the MBCO LRTs to the models with categorical mediator or outcome variables. Such an extension would require defining and estimating indirect effect in the potential outcome framework. A second area is the extension of the MBCO LRTs to a multilevel mediation framework. Finally, we did not consider the missing data. More work is needed to assess the performance of the MBCO LRTs when missing data is present.

## Open Practices Statement

The datasets generated and analyzed during the current study as well as the R script and description of code to perform all three MBCO LRTs are available in the figshare repository, https://doi.org/10.6084/m9.figshare.9642953.

## Data Availability Statement

All datasets generated for this study are included in the article/Supplementary Material.

## Author Contributions

DT conceptualized and designed the study, performed the statistical analysis and simulations, and wrote the manuscript.

### Conflict of Interest

The author declares that the research was conducted in the absence of any commercial or financial relationships that could be construed as a potential conflict of interest.

## Footnotes

^1^The datasets generated and analyzed during the current study as well as the R script and description of code to perform all three MBCO LRTs are available in the figshare repository, https://doi.org/10.6084/m9.figshare.9642953

^2^A set difference between two sets **Θ** and **Θ**_0_, denoted by **Θ**\**Θ**_0_, is set consists of all the elements in **Θ** that are not elements in **Θ**_0_.

^3^One could also use the more restricted parameter space under the alternative hypothesis. The two formulations would yield similar results in most practical situations, whereas the formulation adopted in the current study is more commonly used in practice (Wasserman, 2004).
