# Supplementary material for: Bootstrap Model-Based Constrained Optimization Tests of Indirect Effects
Source: Front Psychol. 2020 Jan 20;10:2989. doi: 10.3389/fpsyg.2019.02989 (PMC6984355; doi:10.3389/fpsyg.2019.02989)
Supplement: Supplementary file 1 [file Table_1.DOCX]

Supplemental Materials

In this document, we present a sample of the R code used in the simulation studies using a simulated data. We present the mbco function to perform asymptotic MBCO LRT, semi-parametric bootstrap MBCO LRT, and parametric bootstrap MBCO LRT. We also present the code to compute percentile and bias-corrected bootstrap, profile-likelihood, and Monte 95% Carlo confidence interval (CI). The R code to perform three variations of the MBCO LRT is contained in the file named mbco.R. Before using the mbco function, you need to have the packages OpenMx, RMediation, and modelr installed. Then you need to load the two packages and the R script file mbco.R as follows:

library(OpenMx)
library(RMediation)
library(modelr)
source("mbco.R")

# The mbco function

In this section, we explain the arguments to the mbco function. We also explain the output for the mbco function.

mbco(
 h0 = NULL,
 h1 = NULL,
 R = 10L,
 type = "asymp",
 alpha = 0.05,
 checkHess = "No",
 checkSE = "No",
 optim = "SLSQP",
 precision = 1e-09
)

## Arguments

Below, we explain the arguments for the mbco function.

- h0: An OpenMx model estimated under a null hypothesis, which is a more constrained model
- h1: An OpenMx model estimated under an alternative hypothesis, which is a less constrained model. This is usually a model hypothesized by a researcher.
- R: The number of bootstrap draws.
- type: If 'asymp' (default), the asymptotic MBCO chi-squares test comparing fit of h0 and h1. If 'parametric', the parametric bootstrap MBCO chi-squared test is computed. If 'semi', the semi-parametric MBCO chi-squared is computed.
- alpha: Significance level with the default value of .05
- checkHess: If 'No' (default), the Hessian matrix would not be calculated.
- checkSE: if 'No' (default), the standard errors would not be calculated.
- optim: Choose optimizer available in OpenMx. The default optimizer is "SLSQP". Other optimizer choices include "CSOLNP" and "NPSOL". See mxOption for more details.
- precision: Functional precision. The default value is set to 1e-9. See OpenMx::mxOption for more details.

## Output

A list that contains

- chisq: asymptotic chi-squared test statistic value
- df: chi-squared degrees of freedom
- p: chi-squared p-value computed using the null sampling distribution estimated from the method specified by the argument type

# Example

Below we present an example to show how to use the mbco function to conduct asymptotic, semi-parametric, and parametric MBCO LRT to test an indirect effect for a sequential two-mediator model using a simulated data set. We also present the code to compute percentile and bias-corrected bootstrap, profile-likelihood, and Monte Carlo 95% CI. Below is the code to simulate data using a sequential two-mediator population model.

set.seed(1234)
n <- 100L
beta1 <- 0.39
beta2 <- 0.39
beta3 <- 0.39
x <- rnorm(n)
m1 <- beta1 * x + rnorm(n)
m2 <- beta2 * m1 + rnorm(n)
y <- beta3 * m2 + rnorm(n)
tbl <- data.frame(x, m1, m2, y)

## Full Sequential Two-Mediator Model

First, we fit the full model using the OpenMx package, which is a sequential two-mediator model without any restriction on the indirect effect, $\beta_{1}\beta_{2}\beta_{3}$. The main command to specify a model in OpenMx is mxModel. In parentheses for the mxModel function, we specify all the elements of the mediation model separated by commas. For the two-mediator mediator example, the first element is the name of the model: name = "full model". The second argument is the type="RAM" (default) of the model, which stands for *Reticular Action Model*. Next, we specify a vector of the names of the manifest (observed) variables, manifestVars = manifests. Note that the vector manifests contains the names of the observed variables in the data set tbl.

The function mxPath is used to specify regression coefficients, intercepts, as well as variance and covariances between the variables. The function mxPath corresponds to the graphical representation of paths in an SEM. For a regression coefficient, the first argument to mxPath is from that species one or more predictors; the argument to specifies a dependent variable; for brevity, we can specify more than one predictor. The argument arrows is set to 1 indicating a uni-directional arrow (regression coefficient) that originates from each predictor in the argument from to the dependent variable in the argument to. The argument free=TRUE indicates that the paths are freely estimated (i.e., not fixed) and the argument values indicates starting values for the paths. The argument labels assigns names to the coefficients corresponding to the predictors specified in from. We also use mxPath to specify the variance and residual variances. The argument from specifies the names of the variables for which (residual) variances are requested. We do not need to specify the argument to, which means that that it takes the same variables as the ones in the argument from. The argument arrows is set to 2 indicating that the arrows are bi-directional. A bi-directional arrow starting and ending with the same variable denotes a (residual) variance. The arguments free, values, and labels have the same interpretation as for the path coefficients.

The function mxAlgebra is used to specify the indirect effect. The first argument is the product of three coefficients. The second argument name = "ind" assigns a name to the indirect effect. The first argument to mxData() identifies the data set to be analyzed; observed = tbl specifies the name of the data set in R. The second argument type = "raw" indicates that the data set is in the raw format, which means that the data set includes observations on the participants as opposed to being a summary statistic such as a covariance matrix. After specifying the full model, we run (fit) the model using the function mxRun and save the results as fit_full_model. The summary function prints the results of the fitted full model. The following R script specifies the full model.

alpha <- 0.05 # significance level
mxOption(NULL, "Default optimizer", "SLSQP") ## Choosing the optimizer SLSQP
mxOption(NULL, 'Calculate Hessian', 'Yes')

manifests <- c("x", "m1", "m2", "y") # declare the the names of the manifest variables
full_model <- mxModel(
 name = "full model",
 type = "RAM",
 manifestVars = manifests,
 mxPath(
 from = 'one', # 'one' is used to indicate the intercepts for the variables specified in the argument "to"
 to = manifests,
 free = TRUE, # the intercepts are freely estimated
 values = 0, # starting values for the intercepts are set ro zero
 labels = c("mu_x", "int_m1", "int_m2", "int_y") #labels for the intercepts
 ),
 mxPath(
 from = "x", # coefficient from x to m1
 to = "m1",
 arrows = 1, # uni-directional arrow
 free = TRUE,
 values = 0,
 labels = "b1" # label for the coefficient
 ),
 mxPath(
 from = "m1",
 to = "m2",
 arrows = 1,
 free = TRUE,
 values = 0,
 labels = "b2"
 ),
 mxPath(
 from = "m2",
 to = "y",
 arrows = 1,
 free = TRUE,
 values = 0,
 labels = "b3"
 ),
 mxPath(
 from = manifests, # specifying (residual) variances for the manifest variables
 arrows = 2, # 2 indicates that variance
 free = TRUE,
 values = 0,
 labels = c("s2x", "s2m1", "s2m2", "s2y")
 ),
 mxData(tbl, type = "raw"), # assign the data set for model
 mxAlgebra(b1 * b2 * b3, name = "ind") # define the indirect effect
)

fit_full_model <- mxRun(full_model) # fit the full model

## Running full model with 11 parameters

summary(fit_full_model) # print summary of the analysis results

## Summary of full model
##
## free parameters:
## name matrix row col Estimate Std.Error A
## 1 b1 A 2 1 0.36391535 0.10273297
## 2 b2 A 3 2 0.49243395 0.08711089
## 3 b3 A 4 3 0.49847300 0.09526821
## 4 s2x S 1 1 0.99874166 0.14124291
## 5 s2m1 S 2 2 1.05407719 0.14907122
## 6 s2m2 S 3 3 0.90023066 0.12731322
## 7 s2y S 4 4 1.07814081 0.15247439
## 8 mu_x M 1 1 -0.15676181 0.09993763
## 9 int_m1 M 1 2 0.03715418 0.10392364
## 10 int_m2 M 1 3 0.15664134 0.09489663
## 11 int_y M 1 4 -0.02403404 0.10477282
##
## Model Statistics:
## | Parameters | Degrees of Freedom | Fit (-2lnL units)
## Model: 11 389 1137.305
## Saturated: 14 386 NA
## Independence: 8 392 NA
## Number of observations/statistics: 100/400
##
## Information Criteria:
## | df Penalty | Parameters Penalty | Sample-Size Adjusted
## AIC: 359.3047 1159.305 1162.305
## BIC: -654.1065 1187.962 1153.221
## To get additional fit indices, see help(mxRefModels)
## timestamp: 2019-12-14 14:39:22
## Wall clock time: 0.067132 secs
## optimizer: SLSQP
## OpenMx version number: 2.15.5
## Need help? See help(mxSummary)

## Null Sequential Two-Mediator Model

We now fit the null model where the indirect effect is constrained to zero, $\beta_{1}\beta_{2}\beta_{3}=0$. That is, we recast the null hypothesis as a non-linear constraint in the null model. Below is the OpenMx code for the null model.

null_model <- mxModel(model = full_model,
 name = "Constrained Model",
 mxConstraint(ind == 0, name = "ind0")) #defining the non-linear constraint
fit_null_model <- mxRun(null_model)

## Running Constrained Model with 11 parameters

summary(fit_null_model)

## Summary of Constrained Model
##
## free parameters:
## name matrix row col Estimate Std.Error A
## 1 b1 A 2 1 -3.599773e-47 9.374599e-48 !
## 2 b2 A 3 2 4.924335e-01 8.711089e-02
## 3 b3 A 4 3 4.984739e-01 9.526813e-02
## 4 s2x S 1 1 9.987426e-01 1.412443e-01
## 5 s2m1 S 2 2 1.186344e+00 1.677754e-01
## 6 s2m2 S 3 3 9.002296e-01 1.273123e-01
## 7 s2y S 4 4 1.078140e+00 1.524744e-01
## 8 mu_x M 1 1 -1.567618e-01 9.993711e-02
## 9 int_m1 M 1 2 -1.989387e-02 1.089200e-01
## 10 int_m2 M 1 3 1.566415e-01 9.489625e-02
## 11 int_y M 1 4 -2.403390e-02 1.047716e-01
##
## Model Statistics:
## | Parameters | Degrees of Freedom | Fit (-2lnL units)
## Model: 11 390 1149.126
## Saturated: 14 387 NA
## Independence: 8 393 NA
## Number of observations/statistics: 100/401
##
## Constraint 'ind0' contributes 1 observed statistic.
##
## Information Criteria:
## | df Penalty | Parameters Penalty | Sample-Size Adjusted
## AIC: 369.1258 1171.126 1174.126
## BIC: -646.8906 1199.783 1165.042
## To get additional fit indices, see help(mxRefModels)
## timestamp: 2019-12-14 14:39:22
## Wall clock time: 0.04313207 secs
## optimizer: SLSQP
## OpenMx version number: 2.15.5
## Need help? See help(mxSummary)

For the null model, we modified the full model by adding the constraint. This is a convenient feature in OpenMx as we can modify an existing model to create a new one, called null_model. The first argument model = full_model specifies the name of the model to be modified. The argument name assigns a name to the new, null model. Finally, the function mxConstraint is used to specify the non-linear constraint $\beta_{1}\beta_{2}\beta_{3}=0$. The first argument to mxConstraint specifies that the indirect effect ind, defined previously in the full model as the product of three coefficients, is constrained to zero. The argument name assigns a name to the non-linear constraint. Next, we run the null model and save the results as fit_null_model. Finally, we print the results of the fitted model.

## MBCO LRTs

We use the mbco function to compute the asymptotic MBCO LRT using the argument type='asymp'. All the arguments to the mbco function are explained above.

mbco(
 h0 = fit_null_model,
 h1 = fit_full_model,
 type = "asymp",
 alpha = alpha
)

## $chisq
## [1] 11.82112
##
## $df
## [1] 1
##
## $p
## [1] 0.0005856249

Next, we compute the parametric bootstrap MBCO LRT. The argument type="parametric" specifies the parametric MBCO LRT.

mbco(
 h0 = fit_null_model,
 h1 = fit_full_model,
 type = "parametric",
 R= 1000L,
 alpha = alpha
)

## $chisq
## [1] 11.82112
##
## $df
## [1] 1
##
## $p
## [1] 0.003

Finally, we compute the semi-parametric bootstrap MBCO LRT. The argument type="semi" indicates the semi-parametric MBCO LRT.

mbco(
 h0 = fit_null_model,
 h1 = fit_full_model,
 type = "semi",
 R= 1000L,
 alpha = alpha
)

## $chisq
## [1] 11.82112
##
## $df
## [1] 1
##
## $p
## [1] 0

## Confidence Intervals

In this section, we compute the 95% confidence intervals using percentile and bias-corrected bootstrap, profile-likelihood, and Monte Carlo method. Below, we use OpenMx to produce the percentile and bias-corrected bootstrap CIs, although one can use the lavaan package to compute them as well, as we did in the simulation studies.

R <- 1000L # the number of bootstrap samples
full_boot <- mxBootstrap(fit_full_model, replications = R)

## Running full model with 11 parameters

# percentile bootstrap
cat("95% percentile bootstrap CI\n")

## 95% percentile bootstrap CI

mxBootstrapEval(b1 * b2 * b3,
 full_boot,
 bq = c(alpha / 2, 1 - alpha / 2),
 method = 'quantile')

## SE 2.5% 97.5%
## [1,] 0.02361729 0.04402596 0.1391377

# bias corrected bootstrap
cat("95% bias-corrected bootstrap CI\n")

## 95% bias-corrected bootstrap CI

mxBootstrapEval(b1 * b2 * b3,
 full_boot,
 bq = c(alpha / 2, 1 - alpha / 2),
 method = 'bcbci')

## SE 2.5% 97.5%
## [1,] 0.02361729 0.04808254 0.1421118

full_model_ci <-
 mxModel(model = full_model,
 name = "Two Mediator Model with CI",
 mxCI("ind", interval = 1 - alpha)) #computing profile-likelihood CI

fit_full_model_ci <-
 mxRun(full_model_ci, intervals = TRUE) # Re-run the model to compute profile-likelihood CI

## Running Two Mediator Model with CI with 11 parameters

summary(fit_full_model_ci) #summary

## Summary of Two Mediator Model with CI
##
## free parameters:
## name matrix row col Estimate Std.Error A
## 1 b1 A 2 1 0.36391535 0.10273297
## 2 b2 A 3 2 0.49243395 0.08711089
## 3 b3 A 4 3 0.49847300 0.09526821
## 4 s2x S 1 1 0.99874166 0.14124291
## 5 s2m1 S 2 2 1.05407719 0.14907122
## 6 s2m2 S 3 3 0.90023066 0.12731322
## 7 s2y S 4 4 1.07814081 0.15247439
## 8 mu_x M 1 1 -0.15676181 0.09993763
## 9 int_m1 M 1 2 0.03715418 0.10392364
## 10 int_m2 M 1 3 0.15664134 0.09489663
## 11 int_y M 1 4 -0.02403404 0.10477282
##
## confidence intervals:
## lbound estimate ubound note
## Two Mediator Model with CI.ind[1,1] 0.03512483 0.08932849 0.1735851
##
## Model Statistics:
## | Parameters | Degrees of Freedom | Fit (-2lnL units)
## Model: 11 389 1137.305
## Saturated: 14 386 NA
## Independence: 8 392 NA
## Number of observations/statistics: 100/400
##
## Information Criteria:
## | df Penalty | Parameters Penalty | Sample-Size Adjusted
## AIC: 359.3047 1159.305 1162.305
## BIC: -654.1065 1187.962 1153.221
## To get additional fit indices, see help(mxRefModels)
## timestamp: 2019-12-14 14:39:22
## Wall clock time: 0.04048395 secs
## optimizer: SLSQP
## OpenMx version number: 2.15.5
## Need help? See help(mxSummary)

We use the ci function in the RMediation package to compute a 95% Monte Carlo CI. The first argument mu for this function is a vector of the coefficient estimates, and the second argument Sigma is a covariance matrix of the coefficient estimates. We use the functions coef and vcov to extract the path coefficients and covariance matrix of the coefficients from the full model, respectively. The argument quant accepts a formula for the indirect effect that starts with the symbol “~”.

ci(coef(fit_full_model),Sigma = vcov(fit_full_model), quant = ~b1*b2*b3)[1:3]

## Warning in vcov.MxModel(fit_full_model): The 'Calculate Hessian' option is disabled. This may result in a poor accuracy vcov matrix.
## Turn on with mxOption(model, 'Calculate Hessian', 'Yes')

## [[1]]
## 2.5 % 97.5 %
## 0.03229844 0.16865880
##
## $Estimate
## [1] 0.0893088
##
## $SE
## [1] 0.03508924
